# Lack of Statin Therapy and Outcomes After Ischemic Stroke: A Population-Based Study

**DOI:** 10.1161/STROKEAHA.122.040536

**Published:** 2023-02-07

**Authors:** Julia Åivo, Jori O. Ruuskanen, Aleksi Tornio, Päivi Rautava, Ville Kytö

**Affiliations:** 1 Neurocenter, Department of Neurology, Turku University Hospital and University of Turku, Finland (J.Å., J.O.R.).; 2 Integrative Physiology and Pharmacology, Institute of Biomedicine, University of Turku, Finland (A.T.).; 3 Unit of Clinical Pharmacology, Turku University Hospital, Finland (A.T.).; 4 Department of Public Health, University of Turku, Finland (P.R.).; 5 Turku Clinical Research Centre, Turku University Hospital, Finland (P.R., V.K.).; 6 Heart Center, Turku University Hospital and University of Turku, Finland (V.K.).; 7 Research Center of Applied and Preventive Cardiovascular Medicine, University of Turku, Finland (V.K.).; 8 Center for Population Health Research, Turku University Hospital and University of Turku, Finland (V.K.).

**Keywords:** cardiovascular diseases, infarction, ischemic stroke, risk factors, stroke

## Abstract

**Background:**

Statin treatment is effective at preventing adverse vascular events after ischemic stroke (IS). However, many patients fail to use statins after IS. We studied the impact of not using statins after IS on adverse outcomes.

**Methods:**

IS patients (n=59 588) admitted to 20 Finnish hospitals were retrospectively studied. Study data were combined from national registries on hospital admissions, mortality, cancer diagnoses, prescription medication purchases, and permissions for special reimbursements for medications. Usage of prescription medication was defined as drug purchase within 90 days after hospital discharge. Ongoing statin use during follow-up was analyzed in 90-day intervals. Differences in baseline features, comorbidities, other medications, and recanalization therapies were balanced with inverse probability of treatment weighting. Median follow-up was 5.7 years.

**Results:**

Statin therapy was not used by 27.1% of patients within 90 days after IS discharge, with women and older patients using statins less frequently. The average proportion of patients without ongoing statin during the 12-year follow-up was 36.0%. Patients without early statins had higher all-cause mortality at 1 year (7.5% versus 4.4% in patients who did use statins; hazard ratio [HR], 1.74 [CI, 1.61–1.87]) and 12 years (56.8% versus 48.6%; HR, 1.37 [CI, 1.33–1.41]). Cumulative incidence of major adverse cerebrovascular or cardiovascular event was higher at 1 year (subdistribution HR, 1.36 [CI, 1.29–1.43]) and 12 years (subdistribution HR, 1.21 [CI, 1.18–1.25]) without early statin use. Cardiovascular death, recurrent IS, and myocardial infarction were more frequent without early statin use. Early statin use was not associated with hemorrhagic stroke during follow-up. Lack of ongoing statin during follow-up was associated with risk of death in time-dependent analysis (adjusted HR, 3.03 [CI, 2.96–3.23]).

**Conclusions:**

Lack of statin treatment after IS is associated with adverse long-term outcomes. Measures to further improve timely statin use after IS are needed.

Stroke is one of the leading causes of death and disability worldwide.^1^ Elevated low-density lipoprotein levels are a risk factor for cardiovascular disease and stroke.^2^ Statins reduce the risk of cardiovascular events and death after myocardial infarction (MI)^3^ and the risk of stroke recurrence and major coronary events after transient ischemic attack or stroke.^4^

Although the benefits of statin treatment are less evident in patients without a clinical history of atherosclerotic cardiovascular disease, statins have been reported to reduce the net adverse cerebrovascular and cardiovascular event rate and mortality rates in patients with ischemic stroke (IS) and atrial fibrillation.^5^ Statins also appear to alleviate the progression of cerebral small vessel disease.^6^

However, while antithrombotic medication is widely acknowledged and implemented in the secondary prevention of IS, the role of statins is less well established. This inclarity is reflected in differences in the US and European guidelines where the American Heart Association (AHA)/American Stroke Association (ASA) guideline^7^ only recommends statins in stroke with atherosclerotic etiology, and the European Stroke Organisation (ESO) guideline^8^ recommends statins regardless of etiology. Adherence to statin treatment also remains a challenge.^9^ Poor adherence to statins has been reported in approximately one-third of patients with a history of stroke^10–12^ and is mainly due to suspected adverse events.^13,14^ Concerns that statins may increase the risk of hemorrhagic stroke, especially in patients with previous intracerebral hemorrhage (ICH),^4,15^ may also cause reticence to prescribe statins for stroke patients. Furthermore, long-term data on the impact of not using statins after IS is also limited. We set out to investigate the impact of not using statins after IS on adverse outcomes in a longitudinal, population-based investigation.

## Methods

### Data Availability

By law, we are not permitted to disclose data to third parties. Requests to access the data set may be sent to Findata (https://www.findata.fi).

### Study Design

We studied the impact of not using statin therapy early after IS on 1- and 12-year outcomes. The primary outcome of interest was all-cause death. Secondary outcomes were composite major adverse cerebrovascular or cardiovascular events (MACCEs; recurrent IS, MI, or cardiovascular death), MACCE subcomponents, hemorrhagic stroke, and ICH. Studied outcomes are defined in more detail in the Supplemental Methods.

Consecutive adult IS patients admitted between January 1, 2005 and December 31, 2017 were retrospectively identified from the Care Register for Healthcare in Finland. All neurological wards that treat IS patients in mainland Finland were included in the search (20 hospitals, including 5 university hospitals with neurosurgical capability). IS was identified with *International Classification of Diseases, Tenth Revision (ICD-10*) code I63 as the primary discharge diagnosis.^16^ Only first-time emergency ward admissions during the study period were included.

Cardiovascular medications outside ward treatment are available only from pharmacies by prescription in Finland, and reimbursed medications (including all studied medications) are dispensed for a maximum period of 3-month usage. Usage of prescription medication early after IS was defined as drug purchase within 90 days after hospital discharge.^17^ Ongoing statin use during follow-up was analyzed in 90-day intervals (Supplemental Methods). To include only patients with possibility and necessity to purchase post-IS medications, patients not discharged to home or home-like facilities (including nursing homes), patients with prolonged (>90 days) admission, patients who died within 90 days after IS, and patients with missing data (0.4%) were excluded (Figure S1).

Comorbidities and prescription medication were detected as previously described (Tables S1 and S2).^18^ Sequential hospital and ward transfers after IS were combined as a single admission. Follow-up started 90 days after index event and ended at the latest on December 31, 2018. The median follow-up was 5.7 (IQR, 3.0–8.9) years. Baseline differences were balanced with inverse probability of treatment weighing (IPTW). This article follows the STROBE reporting guideline.^19^

### Data Sources and Permissions

The study data were combined by linking data from national registries on hospital admissions, mortality and causes of death, cancer diagnoses, prescription medication purchases, and permissions for special reimbursements for medications with unique patient identifier (Supplemental Methods). The Care Register for Healthcare in Finland data, prescription drug purchase data, special reimbursement permission data, and Finnish cancer registry data were obtained from the Findata/National Institute for Health and Welfare of Finland (permission no: THL/164/14.02.00/2021). Mortality data were obtained from Statistics Finland (permission no: TK-53-484-20). Included registries are mandatory by law and include full coverage of the Finnish population.^20^ Informed consent and review by the institutional review board were waived by law due to study design, and the participants were not contacted. The legal basis for processing personal data is public interest and scientific research (EU General Data Protection Regulation 2016/679, Article 6(1)€ and Article 9(2)(j); Data Protection Act, Sections 4 and 6).

### Statistical Analysis

Differences between study groups were analyzed with *t* test and χ^2^ tests. The Cochran-Armitage test was used to analyze trends. Effect sizes in patient characteristics between study groups were evaluated by standardized mean differences. Logistic regression was used to create a propensity score for not using early statin after IS based on age, sex, comorbidities (alcohol abuse, anemia, atrial fibrillation, chronic pulmonary disease, coagulopathy, dementia, depression, drug abuse, heart failure, heart valve disease, hypertension, insulin-dependent diabetes, liver disease, malignancy, MI, noninsulin-dependent diabetes, peripheral vascular disease, prior cerebrovascular disease, psychotic disorder, rheumatic disease, and renal failure), recanalization (thrombolysis or thrombectomy), neurosurgical operation, medications used after IS (angiotensin-converting enzyme inhibitor/angiotensin receptor blocker, ADP inhibitors, antihypertensives, dipyridamole, ezetimibe, and oral anticoagulants), treatment in university hospital, and year of IS (2005–2008, 2009–2012, or 2013–2017).

Propensity score was used to calculate stabilized IPTW (Supplemental Methods).^21^ Weighting resulted in balanced study groups (Table 1). Separate propensity scoring and IPTW adjusting were performed for the following subgroups: men, women, patients aged <60, 60–69, 70–79, and ≥80 years, patients with and without thrombolysis or thrombectomy, diabetes, antihypertensive medication, antithrombotic medication, oral anticoagulation, or prior statin. Covariables between the study groups were balanced in all subgroups (standardized mean differences <0.029 for all). Potential residual confounding required to change the result on primary outcome was estimated by calculating the E-value.^22^

**Table 1.**
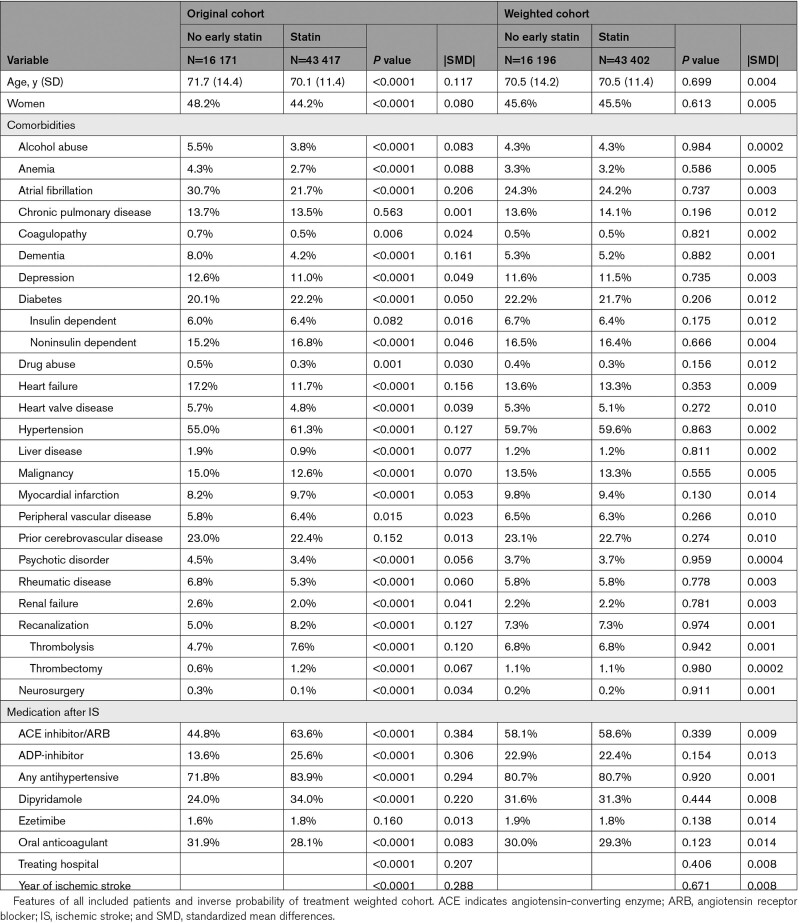
Baseline Features of Patients Without and With Statin Therapy Early After Ischemic Stroke

Primary outcome was studied using Kaplan-Meier method and Cox regression. Incidences of secondary outcomes were studied using cumulative incidence function and Fine-Gray regression notifying competing risk due to non end point-specific death. Additional sensitivity analysis for association of statin use during follow-up with primary outcome was performed on original cohort using multivariable Cox regression with same baseline covariables as used in propensity scoring (except for the year of IS^23^; Supplemental Methods). Robust sandwich-type estimators were used. Results were given as the mean, median, percentage, standardized mean differences, hazard ratio (HR), or subdistribution HR (sHR) with a 95% CI or ±SD. Statistical significance was detected at a *P*-value of <0.05. SAS version 9.4 (SAS Institute Inc, Cary, NC, USA) was used for all analyses.

## Results

**Figure 1.**
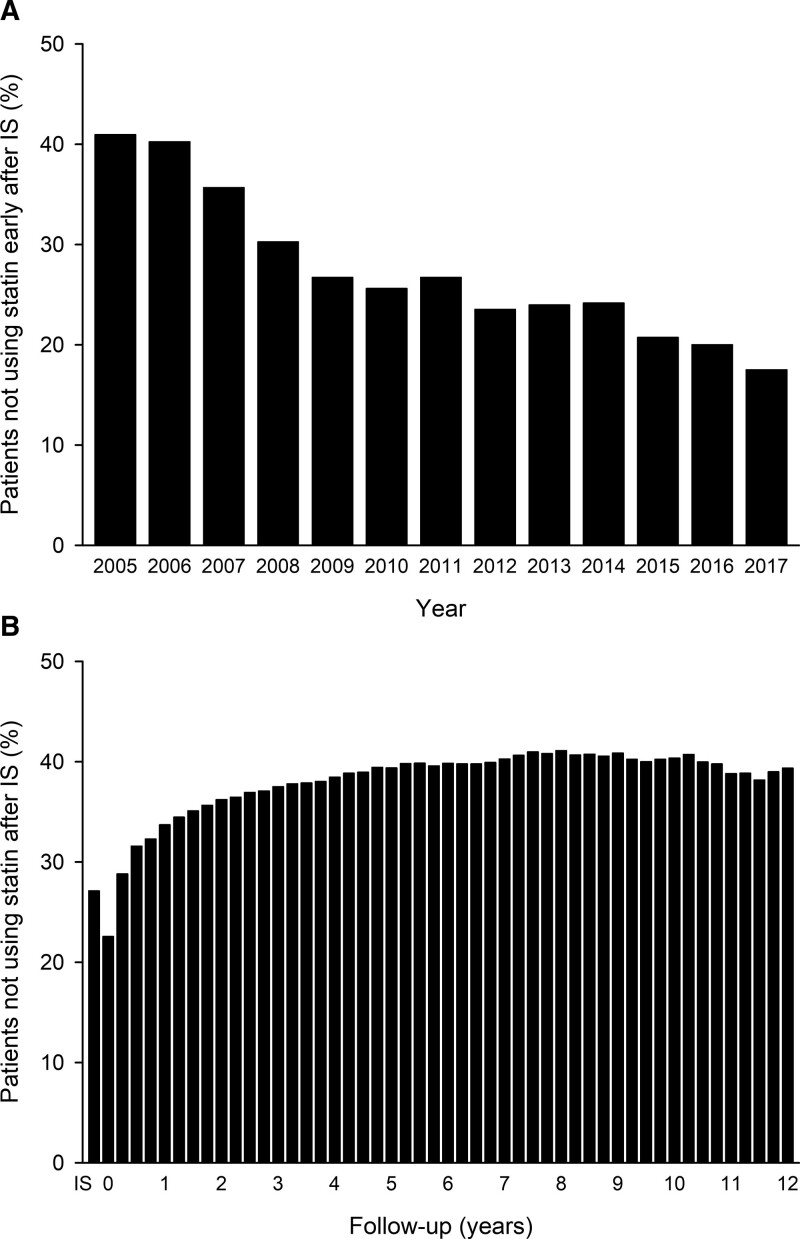
**Usage of statins after ischemic stroke (IS).** Proportion of patients without statin therapy early after IS by the year of IS (**A**). Proportion of patients without statin therapy during the follow-up in 90-d intervals after index IS (**B**).

The study included a total of 59 588 patients (27.1% without statin use early after IS). The proportion of patients without statin therapy early after IS declined from 41.0% in 2005 through 17.5% in 2017 (*P*<0.0001 for trend; Figure 1). The long-term adherence for statins decreased gradually during the first years of follow-up but remained at plateau in longer follow-up (Figure 1). Overall, the average proportion of patients without ongoing statin during the whole 12-year follow-up was 36.0%.

Usage of early statins after IS was less common in women, older patients, and patients with atrial fibrillation, heart failure, rheumatic disease, renal failure, or mental disorder (Table 1). Patients with diabetes and patients with prior MI or peripheral vascular disease as well as patients who underwent acute recanalization therapies used statins more frequently early after IS. Antihypertensive, antiplatelet, and anticoagulant medications were more frequently used by early statin users (Table 1). Study group differences in terms of baseline features, treatments, and usage of other medications were balanced with the IPTW method, resulting in final study groups of 16 196 patients without early statin use and 43 402 patients with statin therapy early after IS (Table 1).

**Figure 2.**
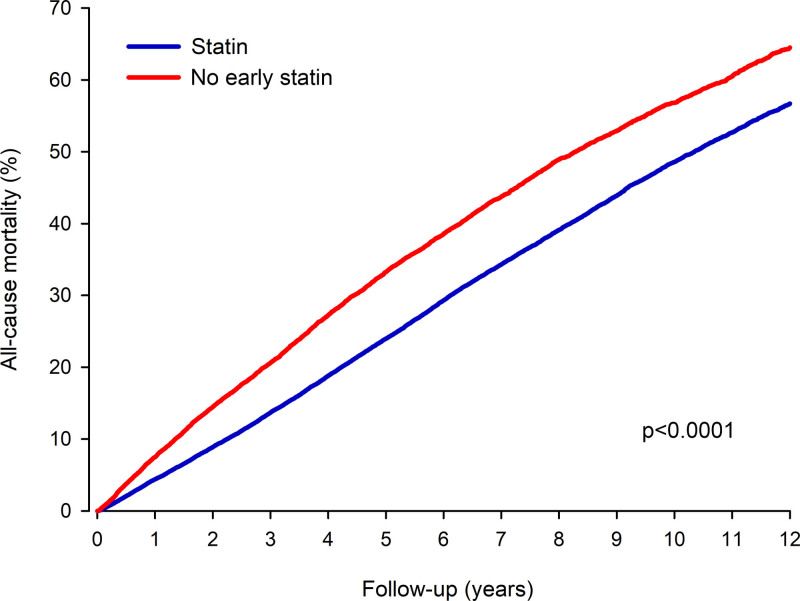
All-cause mortality in inverse probability of treatment weight adjusted patients without and with statin therapy early after ischemic stroke.

During the follow-up period, 21 422 patients (6868 in the no early statin group) died (Figure 2). All-cause mortality was 7.5% in the no early statin group versus 4.4% in the statin group at 1 year (HR, 1.74 [CI, 1.61–1.87]; *P*<0.0001; Table S3). Twelve-year all-cause mortality was 56.8% in the no early statin group versus 48.6% in the statin group (HR, 1.37 [CI, 1.33–1.41]; *P*<0.0001; Table S4). The E-value was 2.08 (CI, 1.99–2.17). In time-dependent analysis, the lack of ongoing statin during follow-up was associated with increased risk of death (HR, 3.03 [CI, 2.96–3.23]; *P*<0.0001).

**Figure 3.**
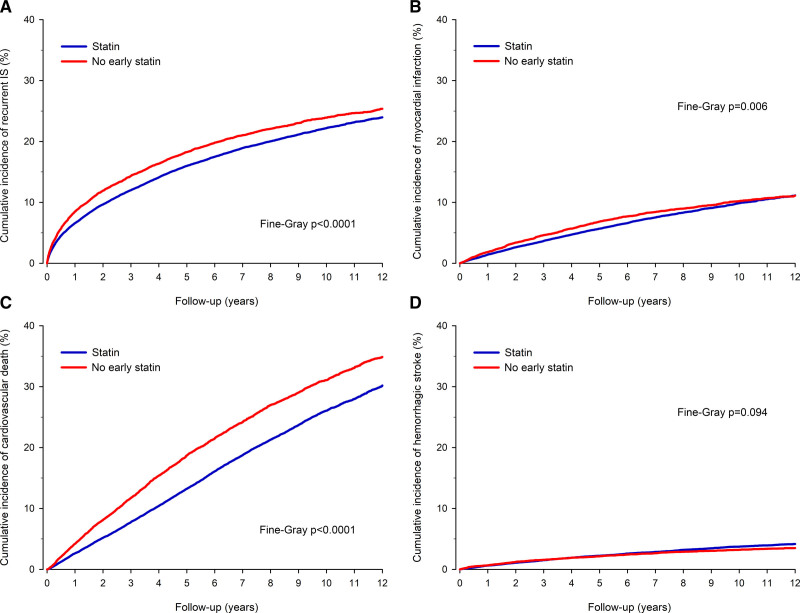
Cumulative incidence of major adverse cerebrovascular or cardiovascular event (MACCE) in inverse probability of treatment weight adjusted patients without and with statin therapy early after ischemic stroke.

**Figure 4.**
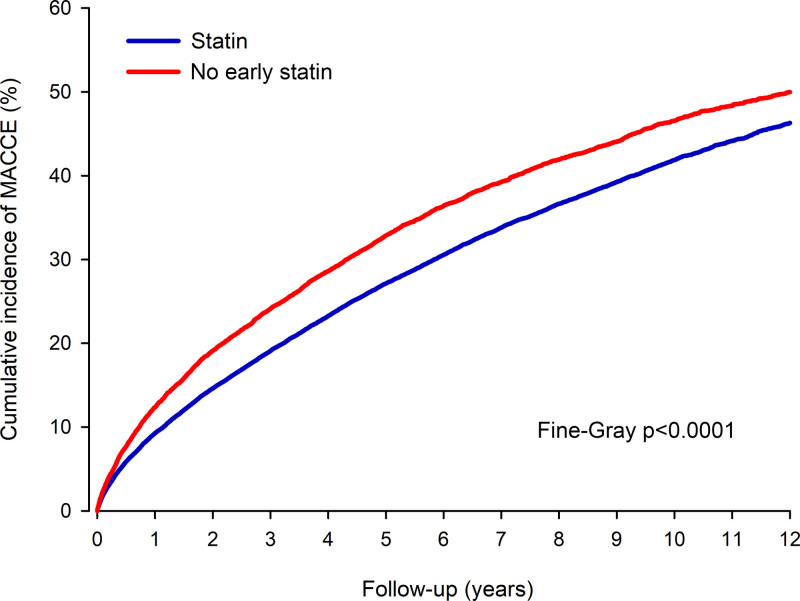
**Secondary outcomes.** Cumulative incidence of (**A**) recurrent ischemic stroke ([IS]; **B**) myocardial infarction, cardiovascular death (**C**), and (**D**) hemorrhagic stroke in inverse probability of treatment weight adjusted patients without and with statin therapy after ischemic stroke.

MACCE occurred in 19 828 patients (6021 in the no early statin group) during the follow-up period (Figure 3). Of all patients, 10 914 had recurrent IS, 4394 had MI, and 11 663 died due to cardiovascular causes, and 1607 suffered hemorrhagic stroke (Figure 4). Cumulative incidence of MACCE was 12.4% in the no early statin group versus 9.3% in the statin group (sHR, 1.36 [CI, 1.29–1.43]; *P*<0.0001) at 1 year. At 12-year follow-up, the cumulative incidence of MACCE was 50.0% in the no early statin group versus 46.3% in the statin group (sHR, 1.21 [CI, 1.18–1.25]; *P*<0.0001).

Cumulative incidence of recurrent IS was 8.4% in the no early statin group versus 6.6% in the statin group at 1 year (*P*<0.0001) and 25.4% versus 24.0%, respectively, at 12 years (sHR, 1.13 [CI, 1.09–1.18]; *P*<0.0001). Cumulative incidence of MI was 1.9% in the no early statin group versus 1.4% in the statin group at 1-year follow-up (Table S3). At the end of the follow-up period, the cumulative incidence of MI was 11.1% in both the study groups (sHR, 1.10 [CI, 1.03–1.17]; *P*=0.006 for the total follow-up). Probability of cardiovascular death was 4.2% in the no early statin group versus 2.6% in the statin group at 1 year (sHR, 1.62 [CI, 1.47–1.78]; *P*<0.0001) and 34.8% versus 30.2%, respectively, at 12 years (sHR, 1.32 [CI, 1.27–1.37]; *P*<0.0001). Cumulative incidence of hemorrhagic stroke was 0.7% in the no early statin group versus 0.6% in the statin group at 1 year, and 3.5% versus 4.2% (sHR, 0.91 [CI, 0.81–1.02]; *P*=0.094) at 12 years. Cumulative incidence of ICH was 2.9% in the no early statin group and 3.3% in the statin group (sHR, 0.93 [CI, 0.82–1.06]; *P*=0.272) at 12 years.

These results were consistent in the subgroup analyses (Table 2). Lack of statin therapy early after IS was associated with increased probability of death and MACCE in patients regardless of sex, age, atrial fibrillation, diabetes, recanalization, or usage of antihypertensive, antithrombotic, or anticoagulant medication (Table 2).

**Table 2.**
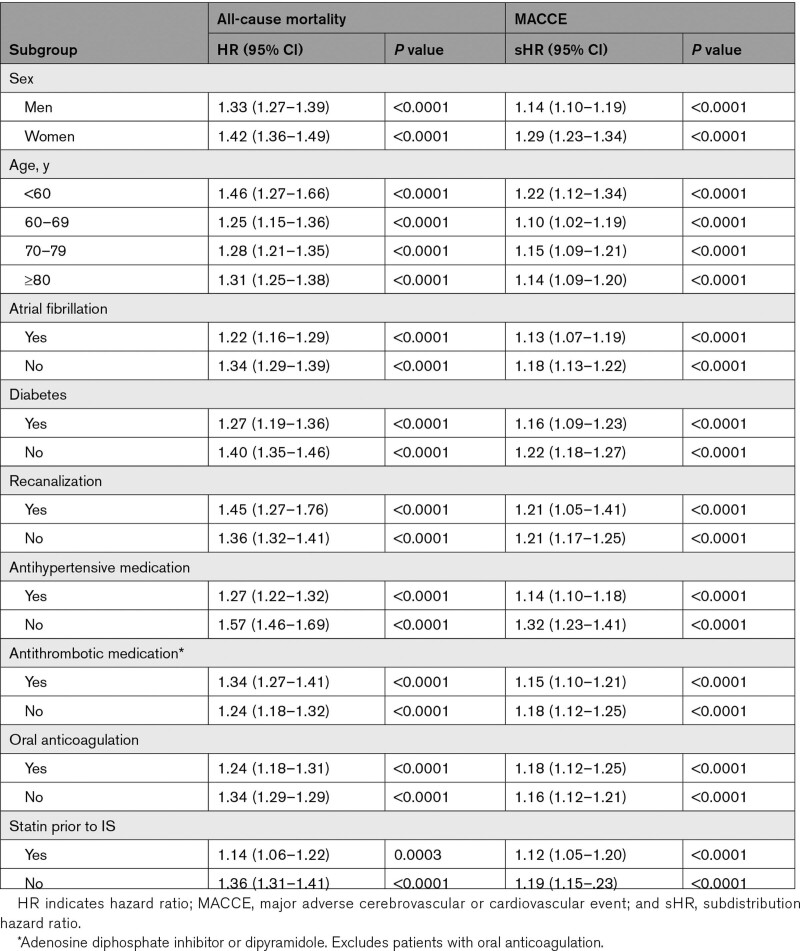
Results of Subgroup Analyses Comparing Long-Term All-Cause Mortality and MACCE Between Patients Not Using Statins Versus Patients Using Statins Early After Ischemic Stroke

## Discussion

This observational, longitudinal, population-based study investigated the outcome impact of lack of statin therapy early after IS. Lack of statins early after IS was associated with increased probability of all-cause death, cardiovascular death, and ischemic events. The risk of death and MACCE were increased in patients not using early statin therapy regardless of sex, age, atrial fibrillation, diabetes, recanalization, or usage of other secondary preventive medications. The risk of ICH did not differ between early statin users and nonusers.

The mechanisms by which statins improve prognosis after ischemic events are multiple. The main effect is the reduction of low-density lipoprotein cholesterol by inhibiting 3-hydroxy-3-methylglutaryl coenzyme A reductase.^24^ Additionally, statins have beneficial pleiotropic effects on the endothelium, immune system, platelets, and vascular smooth muscles.^25^ Statins have been shown to stabilize atherosclerotic plaque^26^ and beneficially effect plaque morphology and volume.^27^ Although the benefits of statin treatment are less evident in patients without a clinical history of atherosclerotic cardiovascular disease, statins have been reported to reduce the net adverse cerebrovascular and cardiovascular event rate and mortality rates in patients with IS and atrial fibrillation.^5^ Statins also appear to alleviate the progression of cerebral small vessel disease.^6^ In a substudy of the SPARCL trial (Stroke Prevention by Aggressive Reduction in Cholesterol Levels), there was no difference in the efficacy of statin treatment regardless of the stroke subtype (large vessel disease, small vessel disease, and stroke of unknown origin).^4^ In a Korean real world data study, statins were effective across stroke subgroups, and also in patients with AF.^10^ In our study, lack of early statin therapy was associated with increased probability of adverse outcomes in patients regardless of sex, age, atrial fibrillation, or diabetes. Our data suggests that early use of statin therapy might be beneficial in all IS patients.

The SPARCL trial established that daily use of high intensity statins in patients with recent stroke or transient ischemic attack led to a 16% reduction in the relative risk of stroke recurrence.^4^ After that, several randomized trials demonstrated the effect of statins on reducing the risk of recurrent stroke and major cardiovascular events in patients with a history of stroke.^28^ Previous observational studies have associated the lack of statin therapy to increased short-term mortality^29,30^ However, large-scale, long-term, follow-up studies of nonstatin users are not, for the best of our knowledge, available. We found that statin usage within the first 90 days after IS was associated with lower all-cause mortality and MACCE at both 1-year and 12-year follow-up. Our long-term results support the previous randomized trials. Differences in outcomes between early statin users and nonusers appeared within first years of follow-up and remained similar during longer follow-up but for MI. The reasons behind observed pattern of cumulative MI incidence during the late follow-up of early statin users (Figure 4) remain unknown, but may relate to potential differences in outcome risk factors of recurrent IS and primary MI.

In our data, 25% of patients did not initiate statin therapy early after IS and 36% lacked ongoing statin during the 12-year follow-up. These results are in line with previous findings. An Italian study showed that 38.9% of IS patients discontinued statin therapy within 12 months after discharge; the mean time from discharge to statin discontinuation was 48.6 days.^31^ In a Korean study (2014–2015), ~35% of patients were nonadherent to statins at 3 and 6 months after IS.^12^ In a Brazilian study, 21.8% of patients received no statins, and 34.9% of patients had poor adherence to statin treatment after IS.^32^

Although the overall adherence to statins seems relatively poor, the proportion of patients who did not use statins early after IS declined from 41% in 2005 through 17.5% in 2017 in our study. There is only limited previous evidence on trends of statin use and adherence. A Korean study demonstrated that the proportion of patients treated with statins after IS increased from 18.3% in 2002 through 63.1% in 2012.^10^ Similarly, in a large retrospective cohort study conducted in the US, in patients with new atherosclerotic cardiovascular disease events, use of statins increased from 50.3% in 2007 through 59.9% in 2016. Patients with coronary heart disease were more likely to receive statins than patients with IS; in 2016, 80.9% of patients with coronary heart disease and 65.8% of patients with IS/transient ischemic attack were using statins.^33^

Antiplatelet and antithrombotic properties of statins have raised concerns that statins might increase the risk of hemorrhagic stroke.^34^ The SPARCL and HPS (Heart Protection Study) trials found increased risk of ICH associated with statin use in patients with previous IS,^4,35^ and, in 2011, a decision analysis of statin therapy in patients with previous ICH concluded that avoiding statin therapy should be considered in patients with a history of ICH.^36^ Later meta-analyses yielded somewhat conflicting results. Two large meta-analyses, including 23 and 33 randomized controlled trials, found no association between statin treatment and ICH.^37,38^ Conversely, an updated meta-analysis found an increased risk of hemorrhagic stroke, especially in patients with prior IS/transient ischemic attack.^39^ A recent large population-based study found no evidence that statins increased the risk of ICH in patients with previous stroke.^40^ In a more recent nationwide case-control study from Denmark, longer duration of statin use was associated with a lower risk of first ever ICH.^41^ Moreover, another large population-based study observed that initiating statin therapy after ICH was associated with a decreased risk of recurrent ICH.^42^ Our results were therefore consistent with this recent real-world evidence of the relative safety of statin therapy regarding the risk of hemorrhagic stroke.

This study has several strengths and limitations. We used nationwide registries with full coverage of the population to avoid selection bias. Results were adjusted with a broad coverage of confounders with propensity matching. Residual confounding may nevertheless influence the results of the study; for example, socioeconomic status was not directly measured. Patients who were not discharged to home or home-like facilities were excluded from the study. These patients are likely to have a poor prognosis and are unlikely to greatly benefit from secondary preventive medications, but we have no means to study this with the current data. We did not have access to more detailed clinical information, such as cholesterol levels, cognition deficits, smoking status, imaging data, stroke subtype, or stroke mechanism. Also, we did not have data on potential statin contraindications. The E-value suggested that the observed HR of 1.37 in long-term mortality could be explained by an unmeasured confounding associated with both early statin use and death by a risk ratio of ≥2.1-fold each, above and beyond the measured confounders, but weaker confounding could not do so.^22^ Our study was primarily designed to be as treated analysis. In addition to marginal effect estimates based on IPTW analyses, a conventional time-dependent on-treatment analysis showed association with lack of statin during follow-up and primary outcome (death). On-treatment analyses should, however, be interpreted with caution as they do not control for reasons of treatment discontinuation that may cause bias.^43^ Also, we did not have data on changes in covariables during follow-up.

## Conclusions

In this population-based study, approximately one-fourth of patients did not use statins after discharge for IS. Lack of statin therapy had a severe adverse association with the risk of death and MACCE. Risk of death was increased by lack of statin use regardless of age, sex, atrial fibrillation, recanalization, or other secondary preventive medications. Importantly, the risk of ICH did not differ between statin users and nonusers. These results suggest that use of statins might be beneficial in all IS patients regardless of IS subtype and underline the importance of measures to improve timely statin use after IS.

## Article Information

### Sources of Funding

This study was supported by grant funding from the Finnish Foundation for Cardiovascular Research, Finnish Cultural Foundation, the Paulo Foundation, the Paavo Nurmi Foundation, and the Finnish Governmental VTR-funding.

### Disclosures

Dr Ruuskanen has received a scientific consultancy fee (Sandoz), a speaker fee (Merck), travel/congress sponsorship (Bristol Myers Squibb and Bayer), and shares (MedBase, Ltd). Dr Tornio has received scientific consultancy fees (CRST, Aplagon) and speaker fees (Bayer, Pfizer). Dr Kytö has received scientific consultancy fees (AstraZeneca), a speaker fee (Bayer), and travel grants and congress sponsorship (Biotronic and Bayer). The other authors report no conflicts.

### Supplemental Material

Supplemental Methods

Tables S1–S4

Figure S1

## Supplementary Material
