# Supplementary material for: Lack of Statin Therapy and Outcomes After Ischemic Stroke: A Population-Based Study
Source: Stroke. 2023 Feb 7;54(3):781–90. doi: 10.1161/STROKEAHA.122.040536 (PMC10561684; doi:10.1161/STROKEAHA.122.040536)
Supplement: Supplementary file 1 [file str-54-781-s001.pdf]

Supplemental Methods

Data sources:

Following nationwide, mandated-by law registry data were collected and combined with unique patient identifier from all study patients:

- All hospital admissions, all outpatient visits in specialist medical care, and all emergency room admissions (International Classification of Diagnosis / ICD-10) and operational codes (Nordic Classification of Surgical Procedures) collected from the CRHF registry obtained from the National Institute for Health and Welfare of Finland. Available from Jan 1st 2004.
- Cancer data from the Finnish Cancer Registry obtained from the National Institute for Health and Welfare of Finland. Available from Jan 1st 1980.
- Purchases of studied prescription drugs 90 days prior and 90 days after IS admission. Including Anatomical Therapeutic Chemical (ATC)-codes and purchase dates obtained from the Social Insurance Institution of Finland. Available from Jan 1st 2004.
- Statin purchases during the follow-up.
- Drug purchase reimbursement permissions including permission codes and underlying ICD diagnoses obtained from the Social Insurance Institution of Finland. Available from Jan 1st 1964.
- Mortality data including date and causes of death obtained from the Statistics Finland. Available from Jan 1st 2005.

Outcome definitions:

- Major adverse cerebrovascular or cardiovascular event: Recurrent ischemic stroke, myocardial infarction, or cardiovascular death.
- Recurrent ischemic stroke: ICD-10 code I63* as primary diagnosis in new hospital admission or as any cause of death.
- Myocardial infarction: ICD-10 code I21* or I22* as primary diagnosis in new hospital admission or as any cause of death.
- Cardiovascular death: Death with ICD-10 code I* as the underlying cause of death.
- Hemorrhagic stroke: ICD-10 code I60*, I61*, or I62* as primary diagnosis in new hospital admission or as any cause of death.
- Intracerebral hemorrhage: ICD-10 code I61* as primary diagnosis in new hospital admission or as any cause of death.

Statin adherence

Statin adherence was studied in 90 day intervals during the follow-up. Number of patients with statin purchase in each period was detected from the study database. Proportion of patients with statin therapy was calculated as: Number of statin purchasers in period / ((Number of patients at risk in the beginning of period + Number of patients at risk in the end of the period)/2). The average proportion of patients using statin during the whole study period was calculated as: Number of patient periods with statin / Number of total patient periods at risk.

Time-dependent regression analysis

Association of not using statin therapy during the follow-up and death after IS was studied using time-dependent Cox regression analysis on original cohort. Statin use during follow-up was measured by 90-day intervals which is the maximum length for distribution of reimbursed prescription medications (incl. statins) from pharmacies in Finland. Patients were allowed to start or stop statin therapy by each 90-day period without limitation. To account for package size differences and potential hospital admissions, statin users with at least 180-day continuous previous use were defined to discontinue statin treatment in second consecutive 90-day period without statin.

The association of statin use during follow-up with primary outcome (death) was studied using multivariable Cor regression modelling. Statin use during follow-up (by 90-day intervals, defined above) was included in regression model as time-dependent variable. The baseline covariables listed in Table 1 (except for the year of IS) were included in regresission model as adjusting variables. Because comorbidities and usage of other medications were available only at baseline, multivariable Cox modelling was used.

	ICD-10 codes	Prescription medication reimbursement codes	Operational codes
**Alcohol abuse**	F10, E52, G62.1, I42.6, K29.2, K70.0, K70.3, K70.9, T51, Z50.2, Z71.4, Z72.1	309	
**Anemia**	D50.0, D50.8, D50.9, D51-D53	107, 122,175, 376	
**Atrial fibrillation**	I48		
**Cerebrovascular disease**	G45, G46, H34.0, I60–I69		
**Chronic pulmonary disease**	I27.8, I27.9, J40–J47, J60–J67, J68.4, J70.1, J70.3	203, 210, 284, 304, 348, 356	
**Coagulopathy**	D65–D68, D69.1, D69.3– D69.6	126, 161, 172, 194, 332, 357, 375	
**Dementia**	A81.0, F00–F03, F05.1, G30, G31	307	
**Depression***	F20.4, F31.3–F31.5, F32, F33, F34.1, F41.2, F43.2		
**Diabetes****	E10-E14	103, 215, 160, 162, 358, 171, 177, 285, 346, 371, 382	
**Drug abuse**	F18, F19, Z715, Z722	339	
**Heart failure**	I09.9, I11.0, I13.0, I13.2, I25.5, I42.0, I42.5–I42.9, I43, I50, P29.0	201, 283, 354, 381	
**Heart valve disease**	A52.0, I05–I08, I09.1, I09.8, I34–I39, Q23.0–Q23.3, Z95.2– Z95.4		
**Hypertension**	I10-I13, I15	205	

**Liver disease**	B18, I85, I86.4, I98.2, K70, K71.1, K71.3– K71.5, K71.7, K72– K74, K76.0, K76.2– K76.9, Z94.4	
**Malignancy**	C	115, 116, 128, 130, 155, 156, 158, 159, 163, 167, 168, 169, 173, 176, 180, 184, 185, 189, 191, 192, 197, 198, 302, 311, 312, 322, 323, 324, 325, 328, 329, 337, 340, 341, 343, 352, 360, 361, 362, 363, 369, 372, 373, 378
**Myocardial infarction**	I21, I22, I25.2	
**Peripheral vascular disease**	I70, I71, I73.1, I73.8, I73.9, I77.1, I79.0, I79.2, K55.1, K55.8, K55.9, Z95.8, Z95.9	
**Prior cerebrovascular disease**	G45, G46, H34.0, I60–I69	
**Psychotic disorder**	F20, F21–F25, F28, F29, F30.2, F31.2, F31.5	112, 188
**Rheumatic disease**	L94.0, L94.1, L94.3, M05, M06, M08, M12.0, M12.3, M30, M31.0– M31.3, M32–M35, M45, M46.0, M46.1, M46.4, M46.8, M46.9	202, 281

**Renal failure**	I12.0, I13.0, N18, N19, N25.0, Z49.0– Z49.2, Z94.0, Z99.2	137, 138, 320	
**Recanalization**			
**Thrombolysis**			AAL10, TPX22
**Thrombectomy**			PA2VT
**Neurosurgery**			AAA27, AAD00, AAD05, AAD10, AAD12, AAD15, AAD16, AAD30, AAD40, AAD42, AAD99, AAF00, AAF05, AAF15, AAF20, AAF25, AAF30, AAF35, AAF40, AAF45, AAF50, AAF99, AAK00, AAK10, AAK20, AAK30, AAK40, AAK80, AAK99

* Or purchase of antidepressant medication within 90 days preceding index admission. ** Or purchase of antidiabetic medication within 90 days preceding index admission.

Table S1. Definitions for baseline co-morbidities and procedures. Co-morbidites were detected from data from index ischemic stroke admission and data available prior to index admission.

	ATC-codes
**ACEi or ARB**	C09, C10BX04, C10BX06, C10BX07, C10BX10-17
**ADP-inhibitor**	B01AC04, B01AC05, B01AC22, B01AC24
**Antidiabetic**	A10
**Antidepressant**	N06A, N06CA
**Antihypertensive**	C02, C03, C07, C08, C09, C10BX03, C10BX04, C10BX06, C10BX07, C10BX10-17
**Dipyramidole**	B01AC07, B01AC30
**Ezetimibe**	C10AX09, C10BA02, C10BA05, C10BA06
**Oral anticoagulant**	B01AA, B01AE, B01AF, B01AX06
**Statin**	C10AA, C10BA, C10BX

Table S2. Anatomical Therapeutic Chemical Classification (ATC) codes for studied prescription medications. ADP = adenosine diphosphate, ACEi = Angiotensin-converting-enzyme inhibitor, ARB = angiotensin receptor blocker.

	1-year IPTW adjusted results			
	Cumulative incidence (%)			
**Outcome**	**No early statin**	**Early statin**	**HR/sHR* (95%CI)**	***P*-value**
**Death**	7.5%	4.4%	1.75 (1.61–1.87)	<0.0001
**MACCE**	12.4%	9.3%	1.36 (1.29–1.43)*	<0.0001
**Recurrent IS**	8.4%	6.6%	1.30 (1.22–1.39)*	<0.0001
**Myocardial infarction**	1.9%	1.4%	1.30 (1.14–1.50)*	0.0002
**Cardiovascular death**	4.2%	2.6%	1.62 (1.47–1.78)*	<0.0001
**Hemorrhagic stroke**	0.7%	0.6%	1.06 (0.85–1.33)*	0.585
**ICH**	0.5%	0.5%	1.09 (0.85–1.41)*	0.485

Table S3. One-year results comparing outcomes between patients not using statins versus patients using statins early after ischemic stroke. ICH = intracerebral hemorrhage, IS = ischemic stroke, IPTW = inverse probability of treatment weight, MACCE = major adverse cerebrovascular or cardiovascular event, sHR = subdistribution hazard ratio.

	12-year IPTW adjusted results			
	**Cumulative incidence (%)**			
**Outcome**	**No early statin**	**Early statin**	**HR/sHR* (95%CI)**	***P*-value**
**Death**	56.8%	48.%	1.37 (1.33–1.41)	<0.0001
**MACCE**	50.0%	46.3%	1.21 (1.18–1.25)*	<0.0001
**Recurrent IS**	25.4%	24.0%	1.13 (1.09–1.18)*	<0.0001
**Myocardial infarction**	11.1%	11.1%	1.10 (1.03–1.17)*	0.006
**Cardiovascular death**	34.8%	30.2%	1.32 (1.27–1.37)*	<0.0001
**Hemorrhagic stroke**	3.5%	4.2%	0.91 (0.81–1.02)*	0.094
**ICH**	2.9%	3.3%	0.93 (0.82–1.06)*	0.272

Table S4. Twelve-year results comparing outcomes between patients not using statins versus patients using statins early after ischemic stroke. ICH = intracerebral hemorrhage, IS = ischemic stroke, IPTW = inverse probability of treatment weight, MACCE = major adverse cerebrovascular or cardiovascular event, sHR = subdistribution hazard ratio.

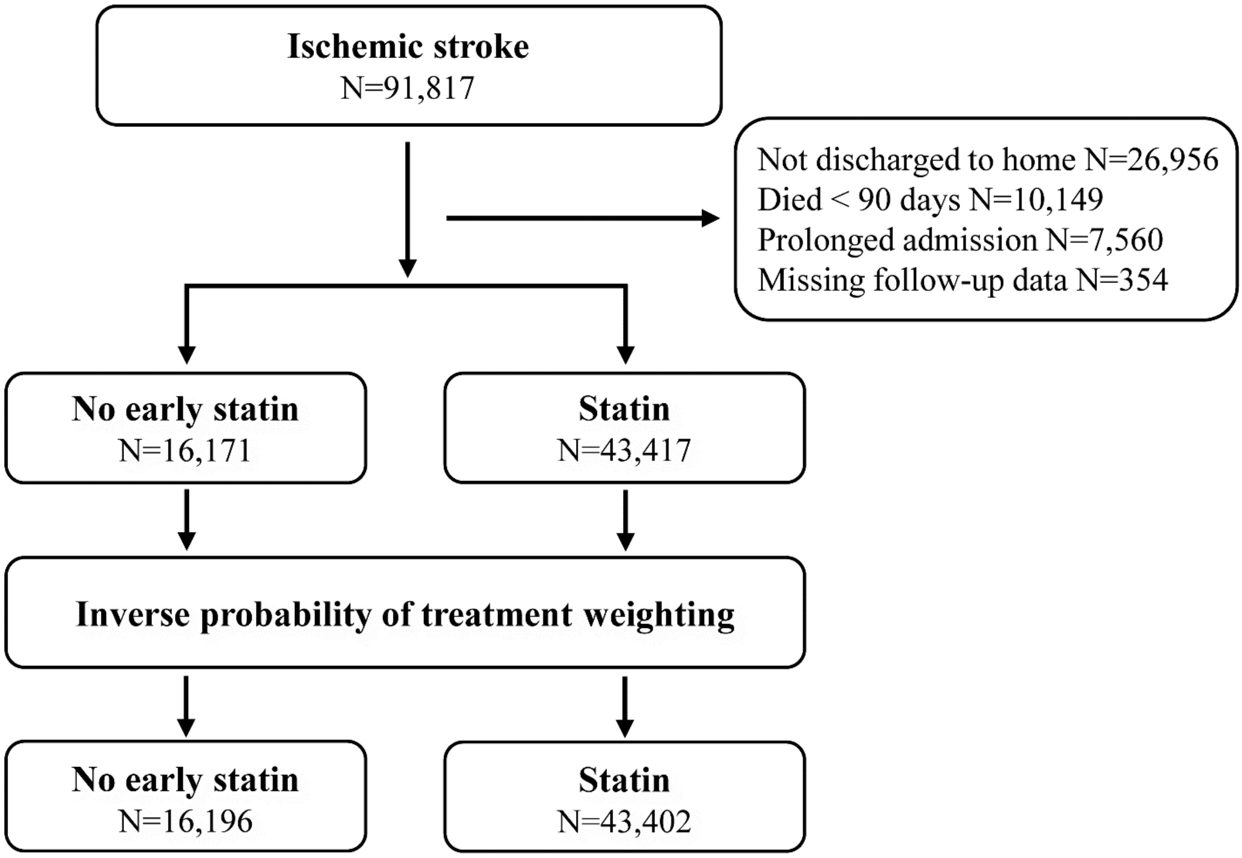

Figure S1. Study flowchart.
